# Sitting time at work and cardiovascular disease risk—a longitudinal analysis of the Study on Mental Health at Work (S-MGA)

**DOI:** 10.1007/s00420-024-02118-3

**Published:** 2025-01-22

**Authors:** Nicole Schmidt, Karla Romero Starke, Martha Sauter, Hermann Burr, Andreas Seidler, Janice Hegewald

**Affiliations:** 1 Institute and Policlinic of Occupational and Social Medicine (IPAS), Faculty of Medicine, Technische Universität Dresden, Fetscherstraße 74, 01307 Dresden, Germany; 2 Division of Work and Health, Federal Institute for Occupational Safety and Health (BAuA), Nöldnerstr. 40-42, 10317 Berlin, Germany

**Keywords:** Sitting position, Seated position, Occupational health, Body mass index, Cardiovascular disease, Longitudinal study

## Abstract

**Purpose:**

This study analyzed longitudinal data to examine whether occupational sitting time is associated with increases in body mass index (BMI) and five-year cardiovascular disease (CVD) risk.

**Methods:**

We included 2,000 employed men and women (aged 31–60) from the German Study on Mental Health at Work (S-MGA) for a BMI analysis and 1,635 participants free of CVD at baseline (2011/2012) for a CVD analysis. Occupational sitting time was categorized into five groups (< 5, 5 to < 15, 15 to < 25, 25 to < 35, and ≥ 35 h per week). BMI change was measured from baseline (2011/2012) to follow-up (2017). Incident CVD included hypertension, heart disease, myocardial infarction, and stroke (all self-reported). Multiple linear regression examined the association between sitting time and BMI change, while modified Poisson regression analyzed CVD incidence, adjusting for age, sex, occupation, shift work, leisure activity, and smoking by sex. Covariates were self-reported.

**Results:**

Over five years, the average BMI change was 0.49 (SD 1.9). We found no association between baseline occupational sitting time and BMI changes, with consistent results in sensitivity analyses. During this period, 245 participants developed cardiovascular disease. There was no increased risk of CVD among those with more sitting time compared to less. No differences in risk were found between women and men.

**Conclusion:**

There was no association between occupational sitting time and five-year changes in BMI or incident CVD.

**Supplementary Information:**

The online version contains supplementary material available at 10.1007/s00420-024-02118-3.

## Introduction

Working adults are sedentary for about 60% of their waking hours, including work time (Prince et al. 2019).Tremblay et al. (2017) define sedentary behavior as “any waking behavior characterized by an energy expenditure ≤ 1.5 metabolic equivalent (METs), while in a sitting, reclining or lying posture”. This applies to various contexts such as work, education, and transportation (World Health Organization (WHO) 2020). Sitting is when weight is supported by the buttocks rather than the feet, with an upright back (Tremblay et al. 2017). It is important to emphasize that sedentary behavior does not mean physical inactivity (World Health Organization (WHO) 2020; Tremblay et al. 2017) and may have physiological consequences that differ from those of a lack of physical activity (PA) (Dunstan et al. 2021; Pinto et al. 2023).

Prolonged periods of being sedentary can negatively impact health outcomes, like quality of life, cognitive function, depression, musculoskeletal pain, and cardiometabolic risk markers (Saunders et al. 2020). Wilmot et al. (2012) found in a systematic review that there was a 147% increased risk of cardiovascular disease (CVD) events in people who were mostly sedentary compared to those who were least sedentary. Long periods of sedentary behaviour are sometimes difficult to counteract with PA (Dunstan et al. 2021; Biswas et al. 2015). A meta-analysis by Bailey et al. (2019) reported an increased risk of CVD with high total daily sitting time, independent of PA.

Sedentary jobs may increase the risk of weight gain (Park et al. 2020), contributing to a higher BMI, particularly in the obesity range (defined as BMI ≥ 30 kg/m^2^), which promotes CVD (Vaduganathan et al. 2022). CVD, including coronary heart disease (CHD) and strokes, is the leading global cause of death (World Health Organization (WHO) 2021). Common cardiologic disorders like heart failure, atrial fibrillation (AF) and ischemic heart disease (IHD) are sex-specific (Gerdts and Regitz-Zagrosek 2019).

The Guideline Development Group identified gaps in the evidence, particularly regarding the health effects of different types and settings of sitting behavior (e.g. sitting at work) (DiPietro et al. 2020). Two systematic reviews found inconsistent results on occupational sitting and health risks, including CVD and cardiometabolic markers (van Uffelen et al. 2010; Reichel et al. 2022). Most studies have focused on overall occupational physical activity (OPA) rather than sitting time specifically (Reichel et al. 2022; van Uffelen et al. 2010; Strippoli et al. 2022; Bonekamp et al. 2023). Reichel et al. (2022) mentioned only one study examining occupational sitting time and CVD incidence (Møller et al. 2016). Among studies on occupational sitting time and BMI, only two provided stratified analyses by sex with mixed results. Eriksen et al. (2015) found a BMI increase in women but not men, whereas Lin et al. (2015) found the opposite.

In 2020, the World Health Organization (WHO) issued its first recommendations on sedentary behavior, urging people to reduce sedentary time and replace it with physical activity of any intensity. The guidelines remain unspecific, with no details on frequency, intensity, or duration (World Health Organization (WHO) 2020). By comparing how different amounts of time spent sitting affect health, more accurate and practical sitting recommendations can be made to prevent specific health risks (Dunstan et al. 2021).

This analysis aims to quantify self-reported sitting time at work to assess its impact on health outcomes. Using longitudinal data from the Study on Mental Health at Work (S-MGA), we investigate whether occupational sitting time is associated with an increase in BMI and five-year CVD risk, while also examining potential sex differences in these associations.

## Methods

### Study design and participants

We used data from the S-MGA, a representative panel survey of employees subject to social security contributions in German. The study was carried out by the Federal Institute for Occupational Safety and Health (BAuA). The survey includes men and women between the ages of 31–60 (Rose et al. 2017). The data for the first two waves were used for analysis (Pattloch et al. 2023).

The first wave of the panel study took place between November 2011 and June 2012 (baseline). A representative sample of 13,590 employees from across Germany were invited to participate. The S-MGA is based on the Employment History, which is a part of the Integrated Employment Biographies (IEB) of the Federal Employment Agency (Oberschachtsiek et al. 2009). This population comprises those subject to social security contributions in Germany (more than 80% of all employees in Germany) who were registered with the Federal Employment Agency on the 31st of December 2010, including those with a mini job earning less than 400 euros per month (Alda et al. 2005). However, self-employed individuals, freelancers, and civil servants are excluded from this population. After a two-step stratified sample (sample points in communities and then using a proportional random selection), a total of 4,511 employees subject to social insurance contributions were surveyed in the first wave of the panel. The response in the first wave was 33%. In the second wave between February and May 2017, *n* = 2,637 (58%) of the people in the first wave were reinterviewed.

**Fig. 1:**
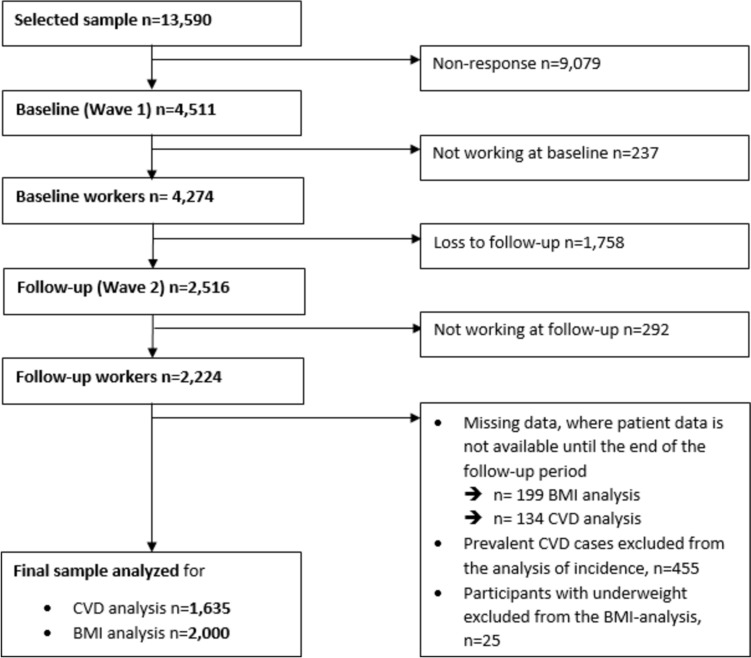
Sample flow chart of participants in baseline (2011/2012) and follow-up (2017)

At baseline, 237 participants were not employed, resulting in 4,274 baseline workers, of whom 1,758 participants were lost to follow-up (Fig. 1). Persons lost to follow-up were less likely to be academics (18%) compared to persons who participated in wave 2 (*n* = 2,516) (24%), and more likely to sit less than 5 h/week (33% vs. 26%). At follow-up, 292 participants were unemployed, resulting in 2,224 participants in the second wave (Fig. 1). In both waves, data were collected face-to-face in the respondents’ homes using computer-assisted personal interviews (CAPI). The survey instruments were calibrated and validated (Rose et al. 2017).

We used a complete case analysis (Pedersen et al. 2017). To evaluate if missing data were Missing Completely at Random (MCAR), the test according to Little was used to estimate distribution parameters (Li 2013). The use of the complete case method is justified because the assumption of complete randomness of the missing values was not rejected (*p* = 0.12) (Hughes et al. 2019). Therefore, we excluded participants with missing information on age, gender, occupational sitting time, occupational level, vigorous LTPA, work shift, smoking, BMI and CVD from our analysis. Respondents reporting zero working hours or no exact specification of weekly working time (e.g., “working hours varied greatly” or “more than 90 h”) were also excluded. This led to the exclusion of 199 participants for BMI analysis and 134 participants for CVD analysis due to missing data. Furthermore, 25 participants with a baseline BMI under 18.5 were excluded because weight loss may differ across the BMI range, and may have another meaning for participants in the underweight range than in the normal or overweight range (Kompaniyets et al. 2023). We also excluded 455 participants who reported prevalent CVD (e.g., high blood pressure, heart disease, heart attack) at baseline for the CVD analysis. The final sample size was 1,635 for the CVD analysis and 2,000 participants for the BMI analysis (Fig. 1).

Information on exposure, outcomes and covariates was self-reported and collected at baseline, and follow-up by answering questions.

### Occupational sitting time

At baseline participants rated how often working in a sitting position applied to their job with five response categories (“never”, “up to a quarter of the time”, “up to half of the time”, “up to three-quarters of the time” or “more than three-quarters of the time/almost always”). Data refer to the main occupational activity, excluding any activity during breaks (Pattloch et al. 2023). The categories of sedentary behavior were assigned five factors (0.000, 0.130, 0.375, 0.625 and 0.875). The proportions indicate the relative size of the category in relation to the total sum of the five factors, which were then multiplied by the weekly working time to calculate the occupational sitting time per week for each person. Similar categorical measures of sitting time at work have been used previously (Eriksen et al. 2015; Møller et al. 2016). Then, the occupational sitting time was grouped into five categories, which were used as the exposure variable: “ < 5 h/week”, “5 to < 15 h/week”, “15 to < 25 h/week”, “25 to < 35 h/week” and “ ≥ 35 h/week”. Møller et al. (2016) used comparable categorical assessments to evaluate sitting time on an ordinal scale during work. To examine if changes in sitting times between the study waves were associated with the change in BMI, a sensitivity analysis was done by examining the change in the reported sitting times. In accordance with Eriksen et al. (2015), the change in occupational sitting time from 2011/2012 to 2017 was calculated by subtracting the sitting time in baseline from the sitting time in follow up. The change was then categorized into three levels: decrease (> 2.5 h/week), no change (− 2.5 to 2.5 h/week) and increase (> 2.5 h/week).

### Body mass index

BMI measures were calculated from weight in kilograms divided by the square of height in meters (kg/m^2^). BMI was provided as whole numbers in the S-MGA dataset. The change in BMI from 2011/2012 to 2017 was calculated by subtracting BMI at baseline from the BMI at follow-up. The difference was expressed as a unit change (e.g., a BMI decrease from 25 to 22 is a −3 unit change). The change in BMI was considered a continuous dependent variable. Similar measures (“change in BMI units”) were used by Bak et al. (2004) and Eriksen et al. (2015).

### Cardiovascular disease

Participants reported their CVD status by answering, “Have you ever been diagnosed with any of the diseases or conditions listed here?” (“yes” or “no”) in 2011/2012 and followed in 2017. A list of diseases and conditions was shown by the interviewer that included “cardiovascular diseases (e.g., hypertension, heart disease, myocardial infarction)”. This list was altered slightly between waves 1 and 2 and stroke was added to the examples of CVD. The question is based on the Work Ability Index (WAI) (Tuomi et al. 1998) by Hasselhorn and Freude (2007).

### Covariates

Confounding variables were identified based on existing knowledge and their potential impact on the relationship between the exposure and outcomes (O'Donoghue et al. 2016), following Directed Acyclic Graphs (DAG) theory (Textor et al. 2016) (Figs. S1, S2). These included sex (Gerdts and Regitz-Zagrosek 2019), age (Rodgers et al. 2019), occupational level (Prince et al. 2019; Thompson et al. 2018), shift work (Torquati et al. 2018), smoking status (Parmar et al. 2023) and LTPA (Prince et al. 2019).

Sex was treated as a categorical term (male or female), with stratified analysis due to gender differences in cardiovascular risk (Walli-Attaei et al. 2020). Age was grouped into 5-year classes based on birth years (1951–1955; 1956–1960; 1961–1965; 1966–1970; 1971–1975 and 1976–1980) at baseline. Baseline occupational level was included as a covariate. Office workers have higher occupational sitting times and lower OPA compared to blue-collar workers (Prince et al. 2019). Occupations involving driving are associated with higher BMIs (Prince et al. 2019). Moreover, occupations impact sitting behavior, influencing physical activity levels and potentially contributing to weight gain (Thompson et al. 2018). Occupational level was classified using the ISCED, based on ISCO 08, and grouped into four categories: “unskilled workers", “skilled workers”, “semi-professionals”, and “academics/managers” (International Labor Office Staff 2012). Unlike ISCED, which does not classify managers, we grouped them with academics, as in other socio-economic classifications (Wirth et al. 2009). This was also done by Conway et al. (2023). Participants reported their smoking status with options ranging from “never smoked” to “currently smoke daily”. For analysis, smoking status was dichotomized into “smoker” and “non-smoker”, with “non-smoker” including those who had never smoked or had quit. LTPA at baseline was included as a confounder, focusing on vigorous physical activities such as running, jogging, cycling, swimming, and ball sports. Participants reported their activity frequency on a scale from “never” to “(almost) daily”. Responses were categorized into three groups: “never/rarely”, “once/several times a week”, and “(almost) daily”, in line with WHO guidelines (2020). Shift workers tend to be less sedentary than non-shift workers (Monnaatsie et al. 2021) but have a higher risk of CVD and increased BMI (Torquati et al. 2018; Łagowska et al. 2024). Participants reported their shift work status at baseline with a “yes” or “no” response.

### Statistical analysis

Study population characteristics at baseline were described using mean and standard deviation (SD) for continuous variables and by frequencies (n) and percentages (%) for categorical variables stratified for BMI categories and CVD at baseline and incidence CVD at follow-up. We also created stratified, sex-specific characteristic tables.

#### BMI analysis

Multiple linear regression models were used to estimate coefficients and 95% CI for the relationship between different levels of occupational sedentary behavior and BMI change over 5 years (2011/12 to 2017). Three models were adjusted for various factors: Model 1 (age, sex), Model 2 (occupational level, shift work), and Model 3 (smoking, leisure-time physical activity), with Model 3 considered as the main analysis. The average BMI change for each sedentary behavior level was estimated using marginal means. Sex-stratified models were also used to examine effect modification (Sargeant et al. 2000; Larsson et al. 2004). Two sensitivity analyses were conducted: one further adjusted for baseline BMI, and the other stratified the data based on changes in sitting behavior to assess their potential impact on the results. This approach was used because changes in sitting behavior over time may affect the outcomes in ways not immediately apparent from the unstratified data. Statistical significance was set at *p* < 0.05 using SPSS version 28 (IBM Corp. 2021).

#### CVD analysis

Poisson regression analysis was performed to determine the incidence rate ratio (IRR) and 95% CI for CVD among individuals with different amounts of occupational sitting time. Three adjustment models were analyzed for main effects using the same adjustments as described above (models 1–3) with the main results interpreted from model 3. To account for overestimated relative risk in Poisson regression with binomial data, robust error variance (sandwich estimation) was applied (Zou 2004). Person-years were used as an offset to adjust for unequal time at risk, assigning 2.5 person-years to those with an incident case and 5 years to those with no incident. Models were stratified by sex to explore potential sex-based variations in the relationship between occupational sitting time and CVD. Statistical significance was set at *p* < 0.05, and SPSS version 28 was used for the analysis (IBM Corp. 2021).

## Results

### Sample

Supplementary Tables S1 and S2 show frequencies and percentages for total BMI at baseline stratified by sex and for total CVD at baseline, CVD events and non-events at baseline stratified by sex. The tables include the distribution of BMI and CVD by age, sex, occupational sitting time, occupational level, shift work, LTPA and smoking behavior. The mean working time across occupational sitting time categories at baseline was also considered to describe potential differences in working hours between the groups (Supplementary Table S3).

#### Sample BMI analysis

The mean BMI of the total sample (n = 2,000) was 26.1 (SD 4.3) with an average age of 46.7 (SD 7.3). There were 38.4% participants in the normal weight range, 43% in the overweight range and 17.4% in the obese range. The average BMI increased over 5 years by 0.49 (SD 1.9). On average, people worked 37.7 h/week (SD 12.3) and spent 17.5 h/week (SD 14.6) sitting. Women (n = 979) had a BMI increase of 0.55 BMI units (SD 2.1) and average occupational sitting time at baseline of 15.2 h/week (SD 13.0). 52.9% of the women sat more than 5 h per week at work. For men (n = 1,021), the BMI increased by 0.43 units (SD 1.7) with a baseline sitting time of 19.7 h/week (SD 15.6). About a quarter of men (26%) sat more than 35 h/week. More information can be found in Supplementary Table S1.

#### Sample CVD analysis

The characteristics of the total sample, incident and non-incident CVD cases are shown in Supplementary Table S2. The mean age for total CVD participants (n = 1,635) was 46.1 years (SD 7.1). After 5 years of follow-up, 245 participants (15%) were diagnosed with cardiovascular diseases (44.9% women and 55.1% men). The mean age of the sample with incident CVD was 47.3 years (SD 6.8) (47.6 years (SD 6.7) for men and 47 years (SD 6.9) for women). On average, the workers with incident CVD sat for 16.4 h per week (SD 14.3) at work. Among women, 10.9% sat more than 35 h at work, compared to 23% of men.

### Association between occupational sitting time and change in BMI

The results of the multiple linear regression analyses investigating the associations between occupational sitting time at baseline and change in BMI are presented in Table 1.

**Table 1:** Association between baseline occupational sitting time in 2011/2012 and five-year change in BMI (*n* = 2,000). The regression coefficient (β) corresponds to the ΔBMI

Occupational sitting time (hours/week)	Change in BMI					
	Model 1^a^		Model 2^b^		Model 3^c^	
	β	*95% CI*	β	*95% CI*	β	*95% CI*
< 5	1	(Ref)	1	(Ref)	1	(Ref)
5 to < 15	−0.12	(−0.36 to 0.13)	− 0.12	(−0.37 to 0.14)	−0.11	(−0.37 to 0.14)
15 to < 25	−0.24	(−0.50 to 0.03)	−0.21	(−0.49 to 0.07)	−0.19	(−0.47 to 0.09)
25 to < 35	−0.12	(−0.39 to 0.15)	−0.09	(−0.39 to 0.20)	−0.09	(−0.39 to 0.20)
≥ 35	−0.28	(−0.53 to −0.02)	−0.24	(−0.53 to 0.05)	−0.23	(−0.52 to 0.06)
Model intercept	0.56	(0.25 to 0.87)	0.59	(0.15 to 1.03)	0.67	(0.21 to 1.13)

BMI, body mass-index; CI, confidence interval; β, regression coefficient B

^a^Model 1: Adjusted for age and sex

^b^Model 2: Adjusted as in model 1 + occupational level and shift work

^c^Model 3: Adjusted as in model 2 + smoking behavior and leisure-time physical activity

The findings indicate that there was no statistically significant association between occupational sitting time and change in BMI (Table 1, Model 3). A non-significant decreased change in BMI was observed in participants sitting more than 35 h (β = −0.23 (95% CI −0.52 to 0.06)) compared to the reference group of participants (Table 1, Model 3).

**Fig. 2:**
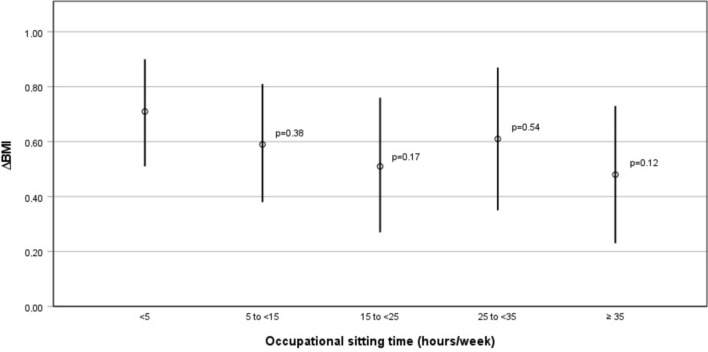
Estimated marginal means* of change in BMI (95% CI) by level of occupational sitting time (hours/week). Based on the regression in Table 1, Model 3^c^. p denotes if means for categories of sedentary behavior from 5 h per week are different from sedentary behavior < 5 h per week. *the means for the ΔBMI variable averaged across every level of the sitting variable. ^c^Model 3: Adjusted for age, sex, occupational level, shift work, smoking behavior and leisure-time physical activity

Figure 2 shows the marginal means of change in BMI (95% CI) estimated by the model 3 for the occupational sitting time groups. When comparing the marginal means of change in BMI, participants in the highest sitting category had a smaller increase in BMI over the 5 years than those in the lowest sitting category.

Figure 2 shows that participants who sat more gained less weight than those who sat for less than 5 h. Individuals who spent less than 5 h sitting had a BMI increase of 0.71 units (95% CI 0.51 to 0.90). Conversely, those who spent more than 35 h per week sitting had an average increase in BMI of 0.48 units (95% CI 0.23 to 0.73, *p* = 0.12).

Further, we performed an additional sensitivity analysis in which we additionally adjusted for baseline BMI, which generated results that were consistent with the main analyses (Supplementary Table S4).

The sex-stratified results show some differences in the change of BMI, but none of the results were statistically significant. Thus, overall, no difference can be ascertained (Table 2).

**Table 2:** Association between baseline occupational sitting time and five-year change in BMI stratified by men (*N* = 1,021) and women (*N* = 979)

Occupational sitting time (hours/week)	Change in BMI			
	men* (n* = *1,021)*		women* (n* = *979)*	
	Model 3^c^		Model 3^c^	
	*β*	*95% CI*	*β*	*95% CI*
< 5	1	(Ref)	1	(Ref)
5 to < 15	−0.21	(−0.53 to 0.12)	−0.00	(−0.40 to 0.39)
15 to < 25	−0.32	(−0.69 to 0.05)	−0.07	(−0.49 to 0.35)
25 to < 35	−0.24	(−0.62 to 0.14)	0.05	(−0.41 to 0.51)
≥ 35	−0.28	(−0.64 to 0.08)	−0.31	(−0.81 to 0.18)
Model intercept	0.41	(−0.25 to 1.07)	0.96	(0.33 to 1.58)

BMI, body mass-index; CI, confidence interval; β, regression coefficient B

^c^Model 3: Adjusted for age, occupational level, shift work, smoking behavior and leisure-time physical activity

In an additional sensitivity analysis, the results were stratified by changes in sitting time. Table 3 presents the association between occupational sitting time and BMI change, with the data stratified into three categories based on changes in occupational sitting time.

**Table 3:** Estimated marginal means* of change in BMI (95% CI) by level of occupational sitting time (hours/week), stratified by change in occupational sitting time (hours/week) between baseline and follow−up. Based on the regression in Table 3, Model 3^c^

Occupational sitting time (hours/week)	Change in BMI and 5-year change in occupational sitting time					
	Model 3^c^		Model 3^c^		Model 3^c^	
	Decrease > 2.5 h/week (*n* = *532*)		No change − 2.5 to 2.5 h/week (*n* = *876*)		Increase > 2.5 h/week (*n* = *511*)	
	*∆*	*95% CI*	*∆*	*95% CI*	*∆*	*95% CI*
< 5	0.56	(−0.21 to 1.32)	0.74	(0.45 to 1.03)	0.65	(0.29 to 1.01)
5 to < 15	0.22	(−0.20 to 0.65)	0.74	(0.40 to 1.07)	0.50	(0.10 to 0.90)
15 to < 25	0.28	(−0.15 to 0.71)	0.46	(0.05 to 0.87)	0.64	(0.19 to 1.10)
25 to < 35	0.60	(0.16 to 1.04)	0.50	(0.04 to 0.97)	0.47	(−0.02 to 0.96)
≥ 35	0.35	(−0.04 to 0.74)	0.64	(0.17 to 1.11)	0.49	(−0.17 to 1.15)

BMI, body mass-index; CI, confidence interval; β, regression coefficient B

*The means for the BMI variable averaged across every level of the sitting variable

^c^Model 3: Adjusted for age, sex, occupational level, shift work, smoking behavior and leisure-time physical activity

No significant association was found between occupational sitting time and change in BMI for any of the three groups (Supplementary Table S5).

The marginal means of BMI change (95% CI) estimated by model 3, stratified by changes in occupational sitting time, suggest a lower BMI increase in individuals who reduced their sitting time by > 2.5 h/week. Specifically, those who reduced their sitting time and sat 5 to < 15 h/week (∆BMI 0.22, 95% CI −0.20 to 0.65) or 15 to < 25 h/week (∆BMI 0.28, 95% CI −0.15 to 0.71) had a smaller BMI increase, while those who maintained or increased their sitting time showed a stable BMI increase across all categories (Table 3, Model 3).

### Association between occupational sitting time and incident CVD

The results of the Poisson regression analysis investigating the associations between occupational sitting time at baseline and incident CVD at follow-up are presented in Table 4.

**Table 4:** Association between baseline occupational sitting time and five-year incident CVD (*N* = 1,635)

Occupational sitting time (hours/week)	CVD cases	Model 1^a^		Model 2^b^		Model 3^c^	
		*IRR*	*95% CI*	*IRR*	*95% CI*	*IRR*	*95% CI*
< 5	67	1	(Ref)	1	(Ref)	1	(Ref)
5 to < 15	50	0.86	(0.60 to 1.24)	0.91	(0.63 to 1.32)	0.94	(0.65 to 1.36)
15 to < 25	49	0.97	(0.67 to 1.40)	1.16	(0.79 to 1.70)	1.20	(0.81 to 1.76)
25 to < 35	36	0.83	(0.55 to 1.24)	1.05	(0.67 to 1.62)	1.04	(0.67 to 1.60)
≥ 35	43	0.78	(0.53 to 1.15)	1.02	(0.66 to 1.58)	1.03	(0.67 to 1.60)

CVD, cardiovascular disease; CI, confidence interval; IRR, incidence rate ratio; Ref, reference category

^a^Model 1: Adjusted for age and sex

^b^Model 2: Adjusted as in model 1 + occupational level and shift work

^c^Model 3: Adjusted as in model 2 + smoking behavior and leisure-time physical activity

There were no statistically significant associations between occupational sitting time and the incidence of cardiovascular disease at the follow-up. Compared to those who sat < 5 h/week, participants who sat 5 to < 15 h/week had a lower CVD risk (IRR 0.94; 95% CI 0.65 to 1.36) and participants who sat 15 to < 25 h/week tended to have higher CVD risk (IRR 1.20; 95% CI 0.81 to 1.76). The IRR for sitting at work 25 to < 35 h/week was 1.04 (95% CI 0.67–1.60), while for sitting ≥ 35 h/week, it was 1.03 (95% CI 0.67 to 1.60). A post-hoc sensitivity analysis was performed by additionally adjusting for self-reported occupational standing time. We did this because long periods of standing are a potential risk factor for cardiovascular disease (Johnsen et al. 2016; Smith et al. 2017; Hall et al. 2019; Bonekamp et al. 2023), and this adjustment could correct inaccurate self-reported sitting times. The results for further adjustments for occupational standing time show higher risks starting at sitting level > 15 h (Supplementary Table S6).

A comparison of the association between occupational sitting time and five-year incident CVD stratified by sex is shown in Table 5.

**Table 5:** Association between occupational sitting time and five-year incident CVD in men (*N* = 776) and women (*N* = 859)

Occupational sitting time (hours/week)	Men		Women	
	Model 3^a^		Model 3^a^	
	*IRR*	*95% CI*	*IRR*	*95% CI*
< 5	1	(Ref)	1	(Ref)
5 to < 15	0.88	(0.53 to 1.46)	1.05	(0.61 to 1.80)
15 to < 25	1.13	(0.64 to 2.00)	1.45	(0.85 to 2.46)
25 to < 35	1.30	(0.72 to 2.34)	0.93	(0.48 to 1.82)
**≥ 35**	1.18	(0.66 to 2.11)	1.06	(0.53 to 2.09)

CVD, cardiovascular disease; CI, confidence interval; IRR, incidence rate ratio; Ref, reference category

^a^Model 3: Adjusted for age, occupational level, shift work, smoking behavior and leisure-time physical activity

No statistical significant differences were observed in the analysis of occupational sitting time on incident CVD stratified by sex.

## Discussion

The aim of this prospective analysis of representative longitudinal data was to investigate if occupational sitting time is associated with an increase in body mass index and the five-year incidence of cardiovascular disease among workers in Germany. We found no association between occupational sitting time at baseline and changes in BMI after five years. On average, the BMI increased over the five years of follow-up. The increases in BMI were smaller in participants with higher occupational sitting times, even after adjusting for confounders such as leisure-time physical activity (∆BMI 0.48, 95% CI 0.23 to 0.73, *p* = 0.21 for sitting ≥ 35 h/week compared to ∆BMI 0.71, 95% CI 0.51 to 0.90 for sitting < 5 h/week). The increase in BMI among workers was observed at all levels of sitting, but this increase was not significantly dependent on the level of sitting. Moreover, there was no increased risk of CVD events in employees engaged in more occupational sitting time compared to employees sitting less after five years of follow-up. In our sex-stratified analysis, we observed no pattern of differences in the risks between females and males.

### Comparison with previous studies

#### Occupational sitting and BMI

We found no association between occupational sitting time and the change in BMI. Our study results are consistent with those of Eriksen et al. (2015), who reported that occupational sitting time at baseline was not significantly associated with five-year BMI change, regardless of sex in a representative sample of the working population in Denmark. The authors calculated the change in BMI like our study, but their analysis of occupational sitting time used different reference groups and other categories of hours per week (low (≤ 10), moderate (< 10 to < 25), high (≥ 25)). We chose to use five categories of weekly occupational sitting time to achieve a better data distribution and to gain additional information about the higher occupational sitting times. Nevertheless, both studies found no associations between occupational sitting time at baseline and changes in BMI.

Similarly, Eriksen et al. (2015) reported that they found no association between baseline occupational sitting time and subsequent changes in BMI in men and women working 20–60 h per week. Eriksen et al. (2015) also examined if changes in sitting time at work were associated with changes in BMI. In our study, this was not a research aim, but we did a sensitivity analysis to see if changes in sitting time between the two surveys modified our results. When the regression model was stratified by changes in sitting time (decrease, no change, increase), the BMI increase tended to be smaller among those who reduced their sitting time by 2.5 h or more and sat less than 25 h/week at work, whereas those who maintained or increased their occupational sitting time showed increases in BMI similar to those observed in the main analysis across all categories (Table 3).

The relationship between occupational sitting and BMI may be influenced by other factors like diet or physical activity. Furthermore, the work style is of importance, as even a few minutes of daily activity in place of sedentary behaviour can lead to improvements in BMI and cardiometabolic biomarkers (Blodgett et al. 2024). The ProPASS study (Blodgett et al. 2024) demonstrates that a work style that incorporates movement, including active breaks or standing work, is also an effective method of reducing health risks and promoting long-term well-being. In a systematic review, Prince et al. (2019) identified workers in desk-based occupations (i.e., office workers) as a population at risk for high levels of sedentary behavior. In the future, this global trend is likely to continue (Woessner et al. 2021). In more industrialized countries, the shift towards sedentary occupations and personal motorized transport is likely to contribute to higher inactivity rates. Conversely, in developing countries, people are more physically active at work and frequently walk or cycle to get from one place to another (Guthold et al. 2018).

In addition, Pinto Pereira and Power (2013), using data of a nationwide cohort, found no trend in BMI gain associated with occupational sitting in mid-adulthood (45–50 years). In comparison to the previous study, we used broader categories for sitting time, thereby enhancing the robustness, stability, and interpretability of the statistical analyses. Also, Pinto Pereira and Power (2013) found that for ages from 45 to 50, each category increase in TV viewing was associated with a 0.11 kg/m^2^ greater BMI increase (95% CI 0.06–0.17). A potential issue is that unhealthy lifestyles may be inversely associated with sedentary work, making it insufficient to control for occupational levels alone to detect the negative effects of sedentary work on BMI (Pinto Pereira et al. 2012). If there was an undetected, albeit weak, adverse effect of occuptional sitting on the cardiovascular system in our study population, it was likely mitigated by lifestyle factors.

Furthermore, no association between occupational sitting time and BMI change was established when using different exposure categories (Saidj et al. 2016) or outcomes like incident obesity (Pulsford et al. 2013). Our results differ somewhat from those of Lin et al. (2015) who found that longer sitting time was associated with higher BMI (BMI increased by 0.216 kg/m^2^ from never sitting to continuously sitting) for the overall sample after eight years of follow-up. Hu et al. (2003) found that each additional two hours of daily sitting at work increased the obesity risk in women by 5% over six years, particularly in those working more than 40 h per week. Thompson et al. (2018) reported a borderline association between high work sitting (90% of time sitting) and BMI increase in women after 6.9 years, with physical activity adjustments slightly reducing the impact on weight gain.

As in all the prospective studies mentioned above, and in this analysis, occupational sitting time was self-reported. Self-report measures generally underestimate sedentary time in comparison to device-based methods (Maes et al. 2020). On average, individuals tended to underestimate their sedentary time by 1.74 h per day (95% CI: −2.11 to −1.38 h/day) in self-report measures when compared to device-based measures (Prince et al. 2020). In addition, single-question measures might result in considerable under-reporting of sitting time compared to all device-based measures (Prince et al. 2020).

#### Occupational sitting and CVD

We found no association between occupational sitting time and incident CVD. The results of our investigation are in line with a cohort study by Møller et al. (2016). There was no difference in the risk of IHD between sedentary time (≥ 25 h/week) and non-sedentary work for either men or women. For each 10-h increase in sitting time at work the RR for IHD was 0.99 (95% CI 0.92–1.06). Møller et al. (2016) mentioned in their discussion that employees with low levels of occupational sitting are likely to have high OPAs, which could have led to the alternative hypothesis of a U-shaped relationship between occupational sitting and IHD. Work often involves prolonged static loading, repetitive and awkward postures, and other physically demanding tasks for several hours daily, typically without adequate recovery time (Holtermann et al. 2018).

Moreover, existing research exhibits substantial heterogeneity across study designs, measures, reference groups, and outcomes (van Uffelen et al. 2010; Reichel et al. 2022). The following studies examined the amount and intensity of physical activity that people engage in during their occupational activities. In a cohort study by Smith et al. (2017) no association was found between sitting at work (type of body posture or movement) and the incidence of heart diseases after 12 years of follow-up. The study found that predominantly standing jobs were associated with twice the risk of heart disease (HR 1.97, 95% CI 0.99–3.90) compared to predominantly sitting jobs. Holtermann et al. (2021) observed that higher OPA was associated with an increased risk of major adverse cardiovascular events. When comparing different exercise postures such as standing and walking, there may be additional variables that influence the results, such as differences in the intensity of the physical activity (Tremblay et al. 2017) and muscle groups used (Dunstan et al. 2021).

Johnsen et al. (2016) found no significant associations between OPA and myocardial infarction. Participants who stood or walked for more than half of their working day had a higher myocardial infarction risk compared to seated workers (OPA total: HR 1.15, 95% CI 0.84–1.58; OPA > 35 h/week: HR 1.20, 95% CI 0.86–1.69). Conversely, those engaged in lifting or carrying during work had a lower myocardial infarction risk than those predominantly seated (OPA total: HR 0.90 (95% CI 0.64–1.28); OPA > 35 h/week: HR 0.98 (95% CI 0.68–1.41)). In their meta-analysis, Kazemi et al. (2024) found OPA had an inverse association with coronary heart disease (CHD) and stroke, but no significant association with CVD, CHD, stroke, or atrial fibrillation when comparing the highest to the lowest OPA categories (CVD HR = 1.01, 95% CI 0.77–1.32; CHD HR = 0.90, 95% CI 0.78–1.04; stroke HR = 0.91, 95% CI 0.80–1.04; atrial fibrillation HR = 1.17, 95% CI 0.99 to 1.38).

We observed a peak in increased risk associated with occupational sitting between 15 to < 25 h per week (IRR 1.20, 95% CI 0.81–1.76). The way people sit and when they sit can be different across different occupations. Certain jobs may involve prolonged and intense periods of sitting, particularly in this specific category (Farrahi et al. 2021). Inaccuracies in self-reported sitting time could also contribute to the peak in increased risk observed in the middle category of sitting time (Prince et al. 2020). In the second wave of the S-MGA, stroke was considered an example of CVD outcomes. This means individuals with prevalent stroke at baseline were likely not excluded, and those experiencing incident stroke may have reported it at follow-up. Stroke survivors differed from non-stroke individuals by spending more time sedentary and less time in light-intensity and moderate-vigorous physical activities (LIPA and MVPA, respectively). Moreover, stroke survivors had fewer and shorter breaks in activity, which were less intense on average compared to non-stroke survivors (Duran et al. 2021). Another explanation for the peak in risk in sitting for 15 to < 25 h/week could be that the workers spend the majority of their remaining working time standing. The time when people are not sitting is spent in another position. Previous studies have investigated an increased risk of CVD for occupational standing (Johnsen et al. 2016; Smith et al. 2017; Hall et al. 2019; Bonekamp et al. 2023) and this could bias the relationship between sitting and CVD. Therefore, we did a sensitivity analysis in which we further adjusted for standing in addition to occupational level and work shift. This additional analysis showed that there were higher risk estimates for sitting in all categories starting at 15 to < 25 h/week with further adjustment for standing at work (results not shown). However, we did not consider the results of this analysis to be primary findings, as artefacts due to measurement issues may have influenced the observed results. There were difficulties in accurately measuring passive standing or active standing (walking) compared to sitting at work. We may have underestimated the effect of sitting because we did not take other postures into account in our analyses due to a lack of information. Further studies should be conducted to better understand different sitting behaviors using standardized self-report or objective measures and including sitting breaks. These surprising results raise new questions, indicating the need for further research to fully understand the complex mechanisms and influencing factors that might explain the negative findings in the present study.

Ferrario et al. (2018) found a statistically significant increase in CHD incidence rates (HR 1.66; 95% CI 1.06–2.59) among male workers with low levels of OPA when compared with the intermediate OPA category, after adjusting for age, cohort, educational level, OPA and sports/physical activity. For cardiovascular disease (CVD), lower OPA levels were associated with a higher risk (HR 1.50; 95% CI 1.01–2.23) compared to the intermediate OPA group, after adjusting for BMI, cholesterol, blood pressure, smoking, diabetes, and alcohol intake.

Allesøe et al. (2015) found no association between sedentary work and IHD risk in female nurses. However, sedentary work was associated with an increased IHD risk among nurses who were inactive or highly active during their leisure-time, after a 15-year follow-up. In their study Allesøe et al. (2016) no significant association was found between sedentary work and elevated IHD risk compared to moderate physical activity at work (HR 1.04; CI 0.77–1.42). However, nurses with hypertension in sedentary jobs had a higher risk (HR 2.33; CI 1.48–3.67), suggesting pre-existing cardiometabolic risk factors may influence cardiovascular disease risk.

#### Occupational sitting with BMI and CVD regarding sex

We did not find notable differences in risks between women and men. The number of studies examining associations between occupational sitting time and BMI or cardiovascular disease risk with sex-stratified analyses is limited. Eriksen et al. (2015) found no associations between categories of occupational sitting in baseline and change in BMI for men and women, respectively. However, they identified a positive association between occupational sitting time and BMI change in women, but not in men, when using occupational sitting time as a quantitative variable. Each unit increase in the occupational sitting category was linked to a BMI increase of 0.13 units (95% CI 0.06–0.20). This suggests the importance of considering occupational sitting time as a quantitative variable rather than a categorical one. An association between longer sitting time at work with higher BMI was found in males by Lin et al. (2015). In women, no association was found. The authors (Lin et al. 2015) noted the potential for residual confounding by not considering the dietary habits of the participants, which could have led to differences between men and women. This may have contributed to the observed gender difference.

In a 15-year follow-up of the Copenhagen City Heart Study, Andersen et al. (2007) found that being sedentary at work had no effect on cardiovascular risk factors such as BMI or blood pressure in both men and women.

### Methodological considerations

Our secondary data analysis possesses several strengths. Firstly, the study employs a prospective research design that can evaluate the temporal relationship between the exposure and outcome of interest. Secondly, the study benefits from a two-stage stratified random population-based representative sample (Rose et al. 2017), enhancing the generalizability of findings. The clearly defined operational definitions of variables also contribute to the clarity and replicability of the study.

However, our study has several limitations. Chronic illnesses were identified as a risk factor for disability pension and unemployment (van Rijn et al. 2014). Therefore, individuals who were out of work at the time of follow-up were excluded, introducing a healthy-worker bias. Moreover, subjects who participated in the follow-up survey may have been generally healthier than those who did not participate. This might have led to a selection bias. Selection bias might also have been present when considering those excluded because of missing data (10% for BMI analysis and 26% for CVD analysis). Another limitation is that we relied on self-reported data for lifestyle factors (LTPA and smoking), outcomes (CVD, weight and height) and occupational factors such as occupational sitting time. They all may have been subject to recall bias, affecting the accuracy of the information. Moreover, the CVD conditions included non-specifically queried outcomes, such as heart disease. As mentioned previously, self-reported measures also typically underestimate sitting duration when compared to methods based on devices (Maes et al. 2020). One major limitation was the use of a single item to estimate the proportion of occupational sitting. It has been shown that single-question measures can result in considerable under-reporting of sedentary behavior compared to objective measures (Prince et al. 2020, 2019). However, device-based objective measurements are difficult to use in population-based studies. Therefore, multi-item questionnaires are realistic alternatives (Prince et al. 2020). For example, the Occupational Sitting and Physical Activity Questionnaire (OSPAQ) has acceptable measurement characteristics for occupational sitting assessment (Maes et al. 2020). As both self-reported and device-based measurements capture important aspects of sedentary behavior, it is recommended that both measures be used for population-based monitoring of sedentary behavior whenever possible (Healy et al. 2011). Furthermore, the study participants estimated their overall sitting times without taking sitting breaks into account. Information on how the breaks are spent would allow a better understanding of how long and how often people sit and what interruptions they make. It is not only the amount of sedentary time that is important, but also how it is accumulated (Farrahi et al. 2021). Better operationalization of sitting breaks, standing and walking in addition to sitting postures is needed for future work.

There were also limitations to the outcomes we evaluated. When BMI is used as an outcome measure, the inability to differentiate between muscle mass and fat mass is an issue (Weir and Jan 2024). Although we tried to consider and control for all known confounding variables, there is a possibility of undetected confounding. This means that other unrecognised or unmeasured factors (e.g., diet) that may be related to exposure and outcome could affect the internal validity of our study. Certain medical conditions or medications can also influence metabolism and result in weight change. The effect of sedentary behavior on weight change is not only related to energy expenditure during physical activity but also to energy intake in regulating energy balance (Júdice et al. 2024). The causes of weight gain could be an increased calorie intake, a change in the composition of the diet, less physical activity and changes in the gut microbiome (Ng and Popkin 2012). Future research should attempt to further minimize these potential confounding effects, possibly by including additional control variables. Despite its limitations, this study contributes to a growing body of literature on sedentary behavior and health, which makes the results of this study important.

## Conclusion

Few studies have examined the relationship between occupational sitting time and changes in body mass index and incidence of cardiovascular disease. These studies focus on the duration and frequency with which people spend sitting during their occupational activities. While our study did not find a significant association with cardiovascular disease, failure to find an effect is as scientifically relevant as finding an effect, and adds to the limited prospective evidence. Our study and existing research suggest there may be no relationship between BMI and occupational sitting. However, unhealthy lifestyle choices may inversely correlate with sedentary work, and controlling for occupational level alone may be insufficient to adjust for the possible impact of work-related lifestyle factors on BMI. These may include, but are not limited to, physical activity levels outside of working hours and dietary habits. Our understanding of how sedentary behavior affects cardiovascular disease events remains incomplete. The findings from our study indicate that researchers face the challenge of including the entire spectrum of physical activities in their analyses by examining the sitting time. Further research is needed to investigate the associations between self-reported or objectively measured postures at work and the risk of cardiovascular disease to understand the mechanisms and optimal levels of sitting in consideration of other postures. As a result, more specific recommendations for workstation design and daily activity patterns may need to be developed.

## Supplementary Information

Below is the link to the electronic supplementary material.

Supplementary file1 (DOCX 516 KB)

## Data Availability

This article is based on the de-facto anonymised data of the Study on Mental Health at Work (S-MGA), waves 1 and 2, version 2, 10.48697/smga.w1w2.suf.2. The data were accessed using a scientific use file, which was made available through the Research Data Centre of the Federal Institute for Occupational Safety and Health (BAuA).

## Abbreviations

AF: Atrial Fibrillation
BAuA: Federal Institute for Occupational Safety and Health
BMI: Body Mass Index
CAPI: Computer-Assistedc Personal Interview
CI: Confidence Interval
CHD: Coronary Heart Disease
CVD: Cardiovascular Disease
DAG: Directed Acyclic Graphs
DALYs: Disability-Adjusted Life Years
IEB: Integrated Employment Biographies
IHD: Ischemic Heart Disease
IRR: Incidence Rate Ratio
ISCED: International Standard Classification of Education
ISCO: International Standard Classification of Occupations
LIPA: Light-Intensity Physical Activity
LTPA: Leisure-Time Physical Activity
MCAR: Missing Completely at Random
MET: Metabolic Equivalent of Task
MVPA: Moderate to Vigorous Physical Activity
OPA: Occupational Physical Activity
OSPAQ: Occupational Sitting and Physical Activity Questionnaire
PA: Physical Activity
SD: Standard Deviation
S-MGA: Study on Mental Health at Work
TIA: Transient Ischemic Attack
WAI: Work Ability Index
WHO: World Health Organization
