# Supplementary material for: Sitting time at work and cardiovascular disease risk—a longitudinal analysis of the Study on Mental Health at Work (S-MGA)
Source: Int Arch Occup Environ Health. 2025 Jan 22;98(1):119–33. doi: 10.1007/s00420-024-02118-3 (PMC11807066; doi:10.1007/s00420-024-02118-3)
Supplement: Supplementary file 1 — Supplementary file1 (DOCX 516 KB) [file 420_2024_2118_MOESM1_ESM.docx]

**Supplementary Material**

**Table S1** Characteristics of participants for BMI-analysis at baseline (2011/2012) by sex

| **Characteristics** | **Total**  **BMI** | |  | **Normal weight**  **BMI ≥18.5 to ≤24.9** **kg/m²** | | | |  | **Overweight**  **BMI 25 to ≤29,9** **kg/m²** | | | |  | **Obesity**  **BMI 30 to**  **≥40** **kg/m²** | | | |
| --- | --- | --- | --- | --- | --- | --- | --- | --- | --- | --- | --- | --- | --- | --- | --- | --- | --- |
|  | ***n=2,000*** | |  | ***n=777*** ***(38.4%)*** | | | |  | ***n= 870*** ***(43%)*** | | | |  | ***n=353 (17.4%)*** | | | |
|  | *N* | *%* |  | *N* | | *%* | |  | *N* | | *%* | |  | *N* | | *%* | |
|  |  |  |  | *m* | *w* | *m* | *w* |  | *m* | *w* | *m* | *w* |  | *m* | *w* | *m* | *w* |
| **Age groups (years)** |  |  |  |  |  |  |  |  |  |  |  |  |  |  |  |  |  |
| 31-35 | 183 | 9.2 |  | 26 | 50 | 10.1 | 9.6 |  | 61 | 19 | 10.8 | 6.2 |  | 15 | 12 | 7.5 | 7.8 |
| 36-40 | 294 | 14.7 |  | 42 | 69 | 16.3 | 13.3 |  | 86 | 51 | 15.2 | 16.7 |  | 25 | 21 | 12.6 | 13.6 |
| 41-45 | 430 | 21.5 |  | 59 | 125 | 22.9 | 24.1 |  | 124 | 56 | 22.0 | 18.3 |  | 37 | 29 | 18.6 | 18.8 |
| 46-50 | 473 | 23.7 |  | 56 | 135 | 21.7 | 26.0 |  | 133 | 75 | 23.6 | 24.5 |  | 49 | 25 | 24.6 | 16.2 |
| 51-55 | 409 | 20.5 |  | 52 | 96 | 20.2 | 18.5 |  | 97 | 71 | 17.2 | 23.2 |  | 53 | 40 | 26.6 | 26.0 |
| 56-60 | 211 | 10.6 |  | 23 | 44 | 8.9 | 8.5 |  | 63 | 34 | 11.2 | 11.1 |  | 20 | 27 | 10.1 | 17.5 |
| **Occupational sitting time (hours/week)** |  |  |  |  |  |  |  |  |  |  |  |  |  |  |  |  |  |
| <5 | 506 | 25.3 |  | 49 | 136 | 19.0 | 26.2 |  | 120 | 96 | 21.3 | 31.4 |  | 46 | 59 | 23.1 | 38.3 |
| 5 to <15 | 440 | 22.0 |  | 49 | 128 | 19.0 | 24.7 |  | 121 | 64 | 21.5 | 20.9 |  | 43 | 35 | 21.6 | 22.7 |
| 15 to <25 | 356 | 17.8 |  | 40 | 111 | 15.5 | 21.4 |  | 93 | 64 | 16.5 | 20.9 |  | 29 | 19 | 14.6 | 12.3 |
| 25 to <35 | 318 | 15.9 |  | 48 | 87 | 18.6 | 16.8 |  | 83 | 44 | 14.7 | 14.4 |  | 35 | 21 | 17.6 | 13.6 |
| ≥35 | 380 | 19.0 |  | 72 | 57 | 27.9 | 11.0 |  | 147 | 38 | 26.1 | 12.4 |  | 46 | 20 | 23.1 | 13.0 |
| **Occupational level (ISCO Skill level Classification)** |  |  |  |  |  |  |  |  |  |  |  |  |  |  |  |  |  |
| unskilled workers | 116 | 5.8 |  | 8 | 31 | 3.1 | 6.0 |  | 28 | 27 | 5.0 | 8.8 |  | 2 | 20 | 1.0 | 13.0 |
| medium skilled workers | 805 | 40.3 |  | 97 | 172 | 37.6 | 33.1 |  | 248 | 119 | 44.0 | 38.9 |  | 111 | 58 | 55.8 | 37.7 |
| higher skilled workers | 562 | 28.1 |  | 62 | 182 | 24.0 | 35.1 |  | 131 | 100 | 23.2 | 32.7 |  | 41 | 46 | 20.6 | 29.9 |
| academics/  managers | 517 | 25.9 |  | 91 | 134 | 35.3 | 25.8 |  | 157 | 60 | 27.8 | 19.6 |  | 45 | 30 | 22.6 | 19.5 |
| **Shift work** |  |  |  |  |  |  |  |  |  |  |  |  |  |  |  |  |  |
| no | 1,668 | 83.4 |  | 211 | 449 | 81.8 | 86.5 |  | 468 | 260 | 83.0 | 85.0 |  | 159 | 121 | 79.9 | 78.6 |
| yes | 332 | 16.6 |  | 47 | 70 | 18.2 | 13.5 |  | 96 | 46 | 17.0 | 15.0 |  | 40 | 33 | 20.1 | 21.4 |
| **Leisure time physical activity** |  |  |  |  |  |  |  |  |  |  |  |  |  |  |  |  |  |
| never | 602 | 30.1 |  | 72 | 124 | 27.9 | 23.9 |  | 133 | 104 | 23.6 | 34.0 |  | 84 | 85 | 42.2 | 55.2 |
| occasionally | 908 | 45.4 |  | 115 | 228 | 44.6 | 43.9 |  | 288 | 138 | 51.1 | 45.1 |  | 87 | 52 | 43.7 | 33.8 |
| often | 490 | 24.5 |  | 71 | 167 | 27.5 | 32.2 |  | 143 | 64 | 25.4 | 20.9 |  | 28 | 17 | 14.1 | 11.0 |
| **Smoking** |  |  |  |  |  |  |  |  |  |  |  |  |  |  |  |  |  |
| non-smoker | 1,418 | 70.9 |  | 173 | 377 | 67.1 | 72.6 |  | 405 | 223 | 71.8 | 72.9 |  | 140 | 100 | 70.4 | 64.9 |
| smoker | 582 | 29.1 |  | 85 | 142 | 32.9 | 27.4 |  | 159 | 83 | 28.2 | 27.1 |  | 59 | 54 | 29.6 | 35.1 |

*Abbreviations: BMI = body mass-index; m = male; w = women*

**Table S2** Characteristics of participants for CVD-analysis at baseline 2011/2012 by sex

| **Characteristics** | **Total**  **CVD** | |  | **Incident CVD** | | | |  | **Non-incident CVD** | | | |
| --- | --- | --- | --- | --- | --- | --- | --- | --- | --- | --- | --- | --- |
|  | ***n=1,635*** | |  | ***n=245 (15%)*** | | | |  | ***n=1,390 (85%)*** | | | |
|  | *N* | *%* |  | *N* | | *%* | |  | *N* | | *%* | |
|  |  |  |  | *m* | *w* | *m* | *w* |  | *m* | *w* | *m* | *w* |
| **Age groups (years)** |  |  |  |  |  |  |  |  |  |  |  |  |
| 31-35 | 160 | 9.8 |  | 6 | 9 | 4.4 | 8.2 |  | 76 | 69 | 11.9 | 9.2 |
| 36-40 | 270 | 16.5 |  | 18 | 11 | 13.3 | 10.0 |  | 112 | 129 | 17.5 | 17.2 |
| 41-45 | 379 | 23.2 |  | 29 | 28 | 21.5 | 25.5 |  | 154 | 168 | 24.0 | 22.4 |
| 46-50 | 394 | 24.1 |  | 40 | 29 | 29.6 | 26.4 |  | 146 | 179 | 22.8 | 23.9 |
| 51-55 | 291 | 17.8 |  | 26 | 22 | 19.3 | 20.0 |  | 103 | 140 | 16.1 | 18.7 |
| 56-60 | 141 | 8.6 |  | 16 | 11 | 11.9 | 10.0 |  | 50 | 64 | 7.8 | 8.5 |
| **Occupational sitting time (hours/week)** |  |  |  |  |  |  |  |  |  |  |  |  |
| <5 | 413 | 25.3 |  | 35 | 32 | 25.9 | 29.1 |  | 132 | 214 | 20.6 | 28.6 |
| 5 to <15 | 349 | 21.3 |  | 25 | 25 | 18.5 | 22.7 |  | 122 | 177 | 19.0 | 23.6 |
| 15 to <25 | 307 | 18.8 |  | 22 | 27 | 16.3 | 24.5 |  | 105 | 153 | 16.4 | 20.4 |
| 25 to <35 | 261 | 16.0 |  | 22 | 14 | 16.3 | 12.7 |  | 105 | 120 | 16.4 | 16.0 |
| ≥35 | 305 | 18.7 |  | 31 | 12 | 23.0 | 10.9 |  | 177 | 85 | 27.6 | 11.3 |
| **Occupational level (ISCO Skill level Classification)** |  |  |  |  |  |  |  |  |  |  |  |  |
| unskilled workers | 88 | 5.4 |  | 6 | 9 | 4.4 | 8.2 |  | 25 | 48 | 3.9 | 6.4 |
| medium skilled workers | 627 | 38.3 |  | 72 | 37 | 53.3 | 33.6 |  | 250 | 268 | 39.0 | 35.8 |
| higher skilled workers | 472 | 28.9 |  | 26 | 41 | 19.3 | 37.3 |  | 155 | 250 | 24.2 | 33.4 |
| academics/  managers | 448 | 27.4 |  | 31 | 23 | 23.0 | 20.9 |  | 211 | 183 | 32.9 | 24.4 |
| **Shift work** |  |  |  |  |  |  |  |  |  |  |  |  |
| no | 1,370 | 83.8 |  | 108 | 85 | 80.0 | 77.3 |  | 532 | 645 | 83.0 | 86.1 |
| yes | 265 | 16.2 |  | 27 | 25 | 20.0 | 22.7 |  | 109 | 104 | 17.0 | 13.9 |
| **Leisure time physical activity** |  |  |  |  |  |  |  |  |  |  |  |  |
| never | 483 | 29.5 |  | 44 | 48 | 32.6 | 43.6 |  | 168 | 223 | 26.2 | 29.8 |
| occasionally | 747 | 45.7 |  | 61 | 45 | 45.2 | 40.9 |  | 316 | 325 | 49.3 | 43.4 |
| often | 405 | 24.8 |  | 30 | 17 | 22.2 | 15.5 |  | 157 | 201 | 24.5 | 26.8 |
| **Smoking** |  |  |  |  |  |  |  |  |  |  |  |  |
| non-smoker | 1,159 | 70.9 |  | 92 | 64 | 68.1 | 58.2 |  | 453 | 550 | 70.7 | 73.4 |
| smoker | 476 | 29.1 |  | 43 | 46 | 31.9 | 41.8 |  | 188 | 199 | 29.3 | 26.6 |

*Abbreviations: CVD = cardiovascular disease; m = male; w = women*

**Table S3** Mean working time across occupational sitting time categories at baseline in both samples

| **Occupational sitting time (hours/week)** | **Weekly Working Hours**  **Sample BMI-analysis** | |  | **Weekly Working Hours**  **Sample CVD-analysis** | |
| --- | --- | --- | --- | --- | --- |
|  | *Mean* | *SD* |  | *Mean* | *SD* |
| **<5** | 30.34 | 13.74 |  | 29.93 | 13.70 |
| **5 to <15** | 35.95 | 12.63 |  | 34.91 | 12.54 |
| **15 to <25** | 37.61 | 10.53 |  | 37.17 | 10.59 |
| **25 to <35** | 41.68 | 7.84 |  | 41.59 | 8.15 |
| ≥**35** | 46.14 | 6.64 |  | 46.15 | 6.48 |


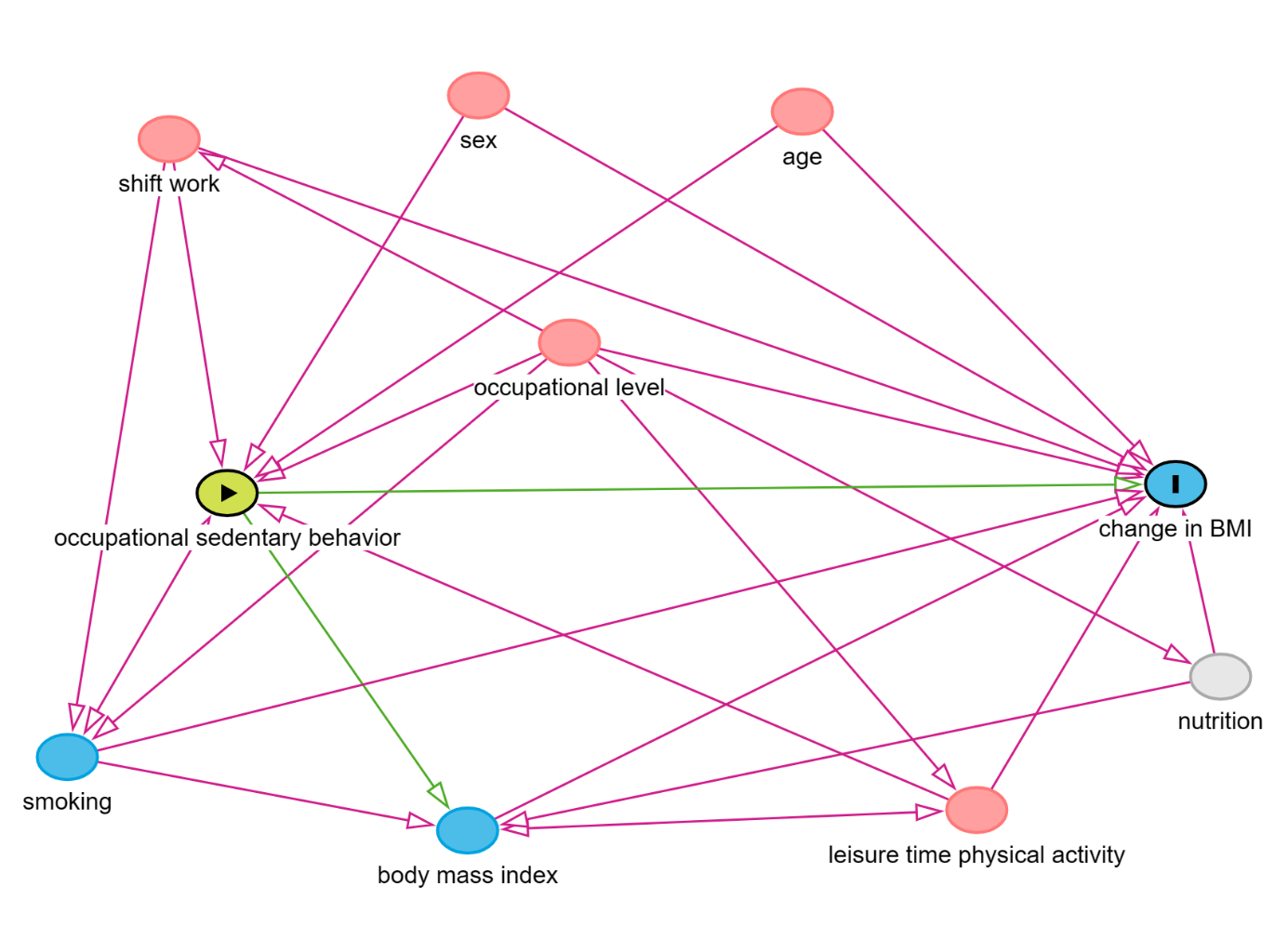


**Fig. S1** Directed acyclic graph showing the relationship between occupational sedentary behavior, BMI changes, and additional causal factors


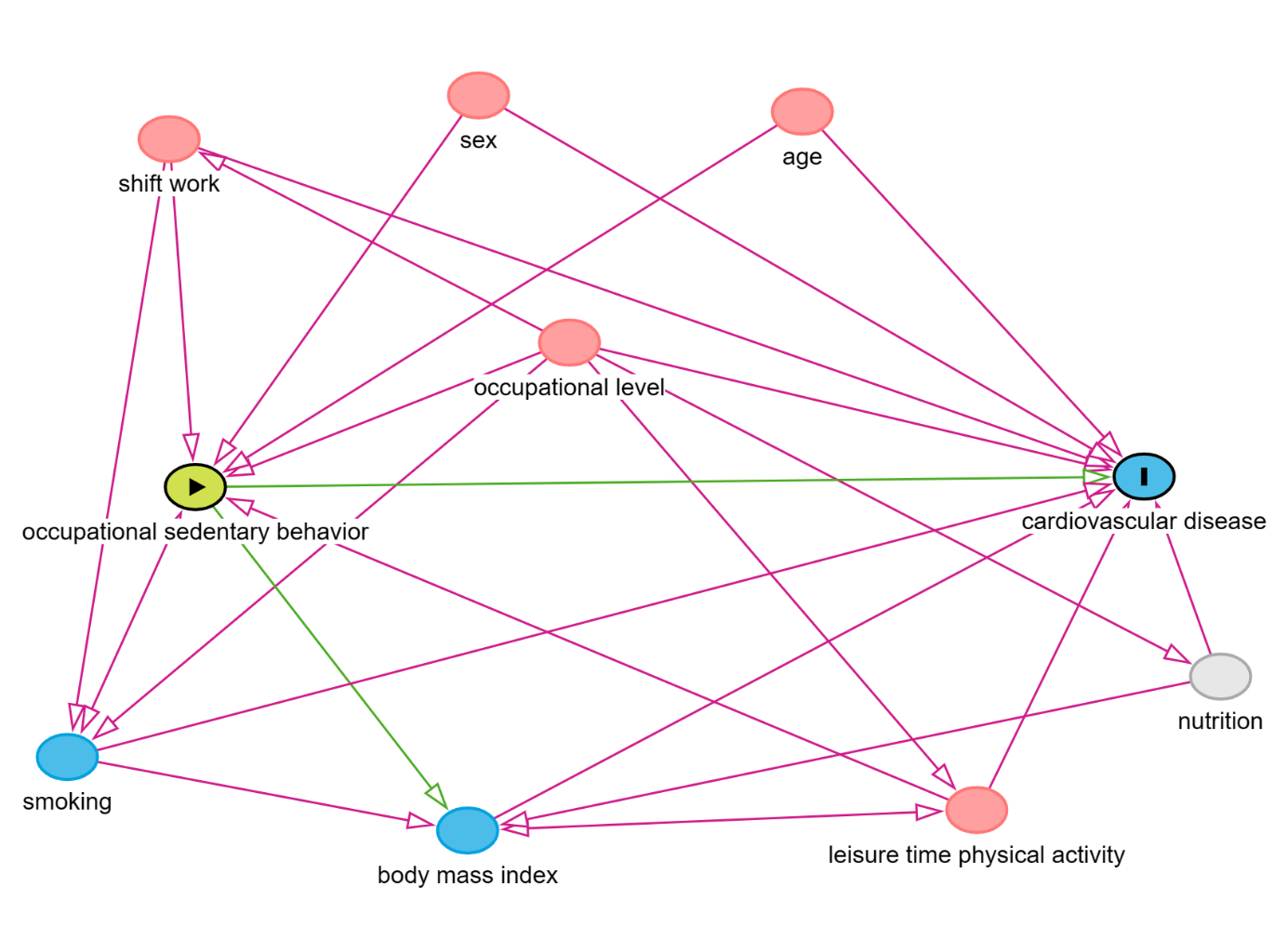


**Fig. S2** Directed acyclic graph showing the relationship between occupational sedentary behavior, cardiovascular disease, and additional causal factors

**Table S4** Association between occupational sitting time in 2012 and five-year change in BMI (N=2,000). The regression coefficient (β) corresponds to the ΔBMI

| **Occupational sitting time (hours/week)** | **Change in BMI** | |
| --- | --- | --- |
|  | **Model 3^c^** | |
|  | *β* | *95% CI* |
| **<5** | 1 | (Ref) |
| **5 to <15** | -0.13 | (-0.38 to 0.13) |
| **15 to <25** | -0.20 | (-0.48 to 0.08) |
| **25 to <35** | -0.11 | (-0.40 to 0.19) |
| ≥**35** | -0.22 | (-0.51 to 0.07) |
| **Model intercept** | 0.82 | (0.36 to 1.29) |

*Abbreviations: BMI = body mass-index, CI = confidence interval, β=regression coefficient B*

^c^ Model 3: Adjusted for age, sex, occupational level, shift work, smoking behavior, leisure time physical activity and baseline BMI (centred).

**Table S5** Association between occupational sitting time and five-year change in BMI stratified by change in occupational sitting time (N=1,919). The regression coefficient (β) corresponds to the ΔBMI

| **Occupational sitting time (hours/week)** | **Change in BMI** | | | | | | | |
| --- | --- | --- | --- | --- | --- | --- | --- | --- |
|  | **Model 3^c^** | |  | **Model 3^c^** | |  | **Model 3^c^** | |
|  | **decrease**  **> 2.5 hours/week**  **(*n=532*)** | |  | **no change**  **−2.5 to 2.5 hours/week**  **(*n=876*)** | |  | **increase**  **> 2.5 hours/week**  **(*n=511*)** | |
|  | β | *95% CI* |  | β | *95% CI* |  | β | *95% CI* |
| **<5** | 1 | (Ref) |  | 1 | (Ref) |  | 1 | (Ref) |
| **5 to <15** | -0.33 | (-1.17 to 0.50) |  | -  0.00 | (-0.40 to 0.39) |  | -0.15 | (-0.59 to 0.30) |
| **15 to <25** | -0.28 | (-1.11 to 0.55) |  | -0.28 | (-0.73 to 0.18) |  | -0.00 | (-0.48 to 0.48) |
| **25 to <35** | 0.49 | (-0.79 to 0.89) |  | -0.23 | (-0.74 to 0.28) |  | -0.18 | (-0.70 to -0.70) |
| ≥**35** | -0.21 | (-1.03 to 0.62) |  | -0.10 | (-0.63 to 0.44) |  | -0.16 | (-0.84 to 0.53) |
| **Model intercept** | 0.57 | (-0.62 to 1.76) |  | 0.95 | (0.25 to 1.65) |  | 0.24 | (-0.71 to 1.19) |

*Abbreviations: BMI = body mass-index, CI = confidence interval, β*=regression coefficient *B*

^c^ Model 3: Adjusted for age, occupational level, shift work, smoking behavior and leisure time physical activity.

**Table S6** Association between occupational sitting time and five-year incident CVD, additionally adjusted for standing (N=1,634)

| **Occupational sitting time (hours/week)** |  |  | **Model 1^a^** | |  | **Model 2^b^** | |  | **Model 3^c^** | |
| --- | --- | --- | --- | --- | --- | --- | --- | --- | --- | --- |
|  | *CVD cases* |  | *IRR* | *95% CI* |  | *IRR* | *95% CI* |  | *IRR* | *95% CI* |
| **<5** | 67 |  | 1 | (Ref) |  | 1 | (Ref) |  | 1 | (Ref) |
| **5 to <15** | 50 |  | 0.86 | (0.60 to 1.24) |  | 0.95 | (0.65 to 1.39) |  | 0.97 | (0.66 to 1.41) |
| **15 to <25** | 49 |  | 0.97 | (0.67 to 1.40) |  | 1.36 | (0.87 to 2.11) |  | 1.38 | (0.89 to 2.15) |
| **25 to <35** | 36 |  | 0.83 | (0.55 to 1.24) |  | 1.25 | (0.75 to 2.08) |  | 1.20 | (0.72 to 2.01) |
| ≥**35** | 43 |  | 0.78 | (0.53 to 1.15) |  | 1.30 | (0.76 to 2.24) |  | 1.29 | (0.75 to 2.21) |

*Abbreviations: CVD = cardiovascular disease, CI = confidence interval, IRR = incidence rate ratio, Ref = reference category.*

^a^ Model 1: Adjusted for age and sex.

^b^ Model 2: Adjusted as in model 1 + occupational level and shift work + occupational standing time.

^c^ Model 3: Adjusted as in model 2 + smoking behavior + leisure time physical activity.
